# Cell Type- and Exposure-Specific Modulation of CD63/CD81-Positive and Tissue Factor-Positive Extracellular Vesicle Release in response to Respiratory Toxicants

**DOI:** 10.1155/2019/5204218

**Published:** 2019-08-14

**Authors:** Frank R. M. Stassen, Pascalle H. van Eijck, Paul H. M. Savelkoul, Emiel F. M. Wouters, Gernot G. U. Rohde, Jacco J. Briedé, Niki L. Reynaert, Theo M. de Kok, Birke J. Benedikter

**Affiliations:** ^1^Department of Medical Microbiology, NUTRIM School of Nutrition and Translational Research in Metabolism, Maastricht University Medical Center, PO box 5800, 6202AZ Maastricht, Netherlands; ^2^Department of Medical Microbiology & Infection Control, VU University Medical Center, P.O. Box 7057, 1007MB Amsterdam, Netherlands; ^3^Department of Respiratory Medicine, NUTRIM School of Nutrition and Translational Research in Metabolism, Maastricht University Medical Center, PO box 5800, 6202AZ Maastricht, Netherlands; ^4^Medical Clinic I, Department of Respiratory Medicine, Goethe University Hospital, Theodor-Stern-Kai 7, 60590 Frankfurt am Main, Germany; ^5^Department of Toxicogenomics, GROW School for Oncology and Developmental Biology, Maastricht University, 6200 MD Maastricht, Netherlands

## Abstract

Chronic exposure to respiratory stressors increases the risk for pulmonary and cardiovascular diseases. Previously, we have shown that cigarette smoke extract (CSE) triggers the release of CD63^+^CD81^+^ and tissue factor (TF)^+^ procoagulant extracellular vesicles (EVs) by bronchial epithelial cells via depletion of cell surface thiols. Here, we hypothesized that this represents a universal response for different pulmonary cell types and respiratory exposures. Using bead-based flow cytometry, we found that bronchial epithelial cells and pulmonary fibroblasts, but not pulmonary microvascular endothelial cells or macrophages, release CD63^+^CD81^+^ and TF^+^ EVs in response to CSE. Cell surface thiols decreased in all cell types upon CSE exposure, whereas depletion of cell surface thiols using bacitracin only triggered EV release by epithelial cells and fibroblasts. The thiol-antioxidant NAC prevented the EV induction by CSE in epithelial cells and fibroblasts. Exposure of epithelial cells to occupational silica nanoparticles and particulate matter (PM) from outdoor air pollution also enhanced EV release. Cell surface thiols were mildly decreased and NAC partly prevented the EV induction for PM_10_, but not for silica and PM_2.5_. Taken together, induction of procoagulant EVs is a cell type-specific response to CSE. Moreover, induction of CD63^+^CD81^+^ and TF^+^ EVs in bronchial epithelial cells appears to be a universal response to various respiratory stressors. TF^+^ EVs may serve as biomarkers of exposure and/or risk in response to respiratory exposures and may help to guide preventive treatment decisions.

## 1. Introduction

The human lungs are covered with a vast epithelial surface, which makes them very efficient for gas exchange, but also highly vulnerable to inhaled exposures [1]. Such exposures include cigarette smoke, as well as gases, volatile compounds, and particulates from outdoor and indoor sources of air pollution. Traffic emissions are major contributors to outdoor air pollution [2] whereas exposure to indoor air pollution is often occupational. For instance, workers of many industrial sectors are exposed to crystalline silica nanoparticles at their workplace [3]. Exposure to respiratory toxicants is associated with several health consequences. Many respiratory exposures contribute to the development or aggravation of pulmonary diseases, such as chronic obstructive pulmonary disease (COPD) [4], (occupational) asthma [5, 6], or pneumoconiosis [7]. Moreover, respiratory exposures are associated with increased risks of lung cancer [8–10] and cardiovascular diseases (CVD) [11–13]. While the cellular and molecular mechanisms underlying the development of respiratory exposure-associated diseases are still incompletely understood, inflammation is known to play an important role.

Epithelial cells form a major cellular target for respiratory exposures as they cover the entire surface of the airways and alveoli [14]. Alveolar macrophages are additional targets due to their localization in the lung lumen. Moreover, both soluble and ultrafine particulate components of inhaled toxicants can translocate across the epithelial barrier or even disturb barrier integrity and interact with cell types located underneath the epithelium, such as fibroblasts and pulmonary microvascular endothelial cells [15–17]. When cells come into contact with environmental stressors, their behaviour is profoundly affected, including the release of extracellular vesicles (EVs) [18, 19]. These EVs are secreted membrane vesicles that carry a complex molecular cargo and exert versatile functions in cell-to-cell communication and in the extracellular space [20]. They are thought to be actively involved in the pathogenesis of several chronic inflammatory diseases, including CVD [21, 22]. We have previously shown that cigarette smoke extract (CSE) increases the amount of small (80-250 nm) CD63^+^CD81^+^ EVs released by bronchial epithelial cells [18]. These CSE-induced EVs were enriched in tissue factor (TF) compared to EVs secreted by unexposed cells [23]. Thus, they likely reflect epithelial activation and damage. Moreover, they exert a TF-dependent procoagulant activity and may thereby contribute to the elevated cardiovascular risk in smokers [23]. We further demonstrated that the EV induction by CSE depended on the oxidative depletion of cellular thiols and could be prevented by antioxidants, such as N-acetyl-L-cysteine (NAC) [18]. In the current study, we aimed to determine whether thiol-dependent EV induction is a universal response to respiratory exposures in different cell types and for different respiratory toxicants. We first investigated the effect of CSE on the EV release by bronchial epithelial cells, pulmonary fibroblasts, macrophages, and pulmonary microvascular endothelial cells. Secondly, we investigated whether respiratory exposures other than CSE also affect EV release by bronchial epithelial cells. For this purpose, cells were stimulated either with particulate matter (PM) from outdoor sources of air pollution or with crystalline silica particles. Total (CD63^+^CD81^+^) and procoagulant (TF^+^) EVs were detected using bead-based flow cytometry, a method that we and others have shown to be suitable for semiquantitative EV measurements [24–26].

## 2. Materials and Methods

### 2.1. Product Information

Detailed product information including catalogue numbers is provided in Supplementary Table 1.

### 2.2. Cell Culture

The bronchial epithelial cell line BEAS-2B (ATCC CRL-9609), the pulmonary fibroblast cell lines MRC-5 (ATCC CCL-171) and HEL-299 (ATCC CCL-137), the monocytic cell line THP-1, and the pulmonary microvascular endothelial cell line HPMEC-ST1.6R (a kind gift from Dr. C.J. Kirkpatrick, Institute for Pathology, University of Mainz, Germany [27]) were maintained at 5% CO_2_ and 37°C. BEAS-2B cells were cultured as described previously [18]. Tests for mycoplasma contamination were performed regularly using the MycoAlert Mycoplasma Detection Kit (Lonza) according to the manufacturer's instructions, and all results were negative. MRC-5 and HEL-299 cells were cultured in a Minimal Essential Medium (MEM; Gibco) supplemented with 1% (*v*/*v*) 100x Non-Essential Amino Acids (Sigma-Aldrich), 2 mM L-glutamine, and 10% (*v*/*v*) foetal calf serum (FCS; Lonza). They were passaged once per week with a 1 : 2 split ratio and used for experiments between passages 20 and 30. THP-1 cells were cultured in RPMI-1640 (Gibco) supplemented with 50 *μ*M *β*-mercaptoethanol (Sigma-Aldrich), 1 mM sodium pyruvate (Sigma-Aldrich), 12.5 mM glucose, and 10% (*v*/*v*) FCS. They were passaged once or twice per week with a split ratio of 1 : 5 to 1 : 20. HPMEC-ST1.6R cells were cultured as described previously in MCDB1 (Gibco) supplemented with 1 *μ*g/ml hydrocortisone (Sigma-Aldrich), 10 ng/ml human recombinant epidermal growth factor (Sigma-Aldrich), 10% (*v*/*v*) FCS, and 1% (*v*/*v*) 100x penicillin and streptomycin (Gibco) [28]. They were subcultured twice per week with a split ratio of 1 : 4 to 1 : 8. Cell culture dishes were precoated with 2% (*v*/*v*) gelatin (Sigma-Aldrich) in MilliQ before HPMEC-ST1.6R cells were seeded.

For experiments, cells were seeded on 12- or 24-well plates. BEAS-2B and HPMEC-ST1.6R were seeded at 5 × 10^4^ cells/cm^2^ and allowed to attach overnight. MRC-5 and HEL-299 cells were seeded at 0.4 × 10^4^ cells/cm^2^ and grown until confluence (usually 72 h). THP-1 cells were seeded at 0.5 × 10^4^ cells/cm^2^ in a growth medium which was additionally supplemented with 200 nM phorbol 12-myristate 13-acetate (PMA; Sigma-Aldrich) to induce monocyte-to-macrophage differentiation. After 72 h, the PMA medium was replaced by a normal growth medium, followed by incubation for another 72 h.

For 2 h prior to exposure, cells were kept in a reduction medium. The reduction medium was DMEM-F12 without phenol-red (Gibco) supplemented with varying percentages of EV-depleted FCS depending on the cell line. For BEAS-2B and HPMEC-ST1.6R cells, the reduction medium was supplemented with 0.1% (*v*/*v*) EV-depleted FCS. For MRC-5 and HEL-299 cells, it was supplemented with 2% (*v*/*v*) EV-depleted FCS, and for THP-1 cells with 2% (*v*/*v*) EV-depleted FCS and 0.5 mM sodium pyruvate. EV-depleted FCS was prepared by 16 h centrifugation at 40,000 rpm (*average* *RCF* = 117,734 × *g*), in a fixed-angle Type 70Ti-rotor in an Optima L-90K preparative ultracentrifuge (Beckman-Coulter, Brea, CA, USA) as described previously [18].

### 2.3. Cell Exposures

CSE, bacitracin, and NAC solutions were prepared as described previously [18]. For CSE production, the smoke of one research reference cigarette without filter (Type 3R4F, Tobacco Health Research, University of Kentucky) was drawn through 2 ml of PBS using a pump at constant speed. CSE was sterile-filtered and used within 15 min after production to avoid loss of unstable or volatile chemical species. CSE was only used when the smoking time was between 6 and 8 minutes and when the delta OD (OD_320 nm_-OD_540 nm_) was between 0.9 and 1.2. Silica particles were obtained from C&E Mineral Corp. and have been characterized previously [29]. Silica suspensions in PBS were prepared as described previously [29]. Particulate matter with an *aerodynamic* *diameter* ≤ 10 *μm* (PM_10_) and ≤2.5 *μ*m (PM_2.5_) was sampled at three primary schools in Maastricht, Netherlands, as described previously [30]. For extraction of PM, filters were cut into pieces of approximately 1 cm^2^ and incubated in 250 ml of dichloromethane overnight at room temperature on a shaker. Dichloromethane was then evaporated using a Rotavap rotational evaporator. The remaining dry material was resuspended in 5 ml methanol and transferred into a centrifuge tube. After 5 minutes of centrifugation at 300 *x g*, the supernatant was transferred into a fresh recipient and evaporated using a Rotavap rotational evaporator. The remaining dry material was resuspended in methanol so that the particulate matter from 200 m^3^ of air was suspended per ml. To obtain a suitable vehicle control, the extraction protocol was also applied to an unused PM_10_ filter. The resulting dry material was weight-matched to the PM_10_ extract and also suspended in methanol.

Prior to exposure, cells were washed twice with PBS followed by the addition of 1 ml of reduction medium. Different concentrations of CSE, NAC, bacitracin (Sigma-Aldrich), silica, PM_10_, and PM_2.5_ or the respective vehicle/solvent controls were then added, followed by 24 h incubation at 37°C and 5% CO_2_. CSE concentrations are given in % (*v*/*v*). Silica concentrations are given in ×10^6^ *μ*m^2^/cm^2^ (surface area of the silica particles/cell culture surface area), 150 × 10^6^ *μm* ^2^/*cm* ^2^ corresponds to 29.4 *μ*g/cm^2^ or 100 *μ*g/ml. PM concentrations are given in m^3^/ml (cubic meters of air filtered to obtain the PM/ml of a cell culture medium). The PM vehicle control was volume-matched to PM_10_. The amount of PM per m^3^ differed between the three sampling locations. Supplementary Table 2 gives a conversion of the PM concentrations used for cell stimulation from m^3^/ml to micrograms per cell culture surface area (*μ*g/cm^2^).

### 2.4. Determination of Cell Metabolic Activity, EV Release, and Exofacial Thiols

Cell viability was assessed by the MTT assay as described previously [18]. Quantification of CD63^+^CD81^+^ EVs by bead-based flow cytometry of conditioned media was also done as described previously [18], except that 200 *μ*l of conditioned media were used instead of 400 *μ*l. For the detection of CD63/CD81/CD9^+^TF^+^ EVs, the protocol was slightly adapted. In brief, 3.5 × 10^8^ beads/ml (4 *μ*m aldehyde/sulphate latex beads 5% (*w*/*v*); Molecular Probes, Life Technologies, Waltham, MA, USA) were coated with a mixture of 42 *μ*g/ml mouse anti-human CD81 antibody (Clone JS-81; BD Biosciences), 42 *μ*g/ml mouse anti-human CD63 antibody (Clone H5C6, BD Biosciences), and 42 *μ*g/ml mouse anti-human CD9 antibody (clone M-L13, BD Biosciences) by overnight incubation in MES buffer. Unconditioned (negative control) or conditioned media (200 *μ*l) were incubated overnight with the beads, followed by staining with 0.01 mg/ml phycoerythrin- (PE-) labelled mouse anti-human TF antibody (Clone HTF-1; BD Biosciences) or PE-labelled IgG1,k isotype control (Clone MOPC-21, BD Biosciences). Measurements were performed on a BD FACSCanto II with FACSDiva V8.0.1 software. The PE-gate was set so that 2% of beads of the unconditioned medium control were PE-positive. Relative fluorescence units were then calculated by multiplying the % of positive beads with the mean fluorescence intensity. Supplementary Figure 1 shows the bead gating strategy (panel A) and the fluorescence intensity of EV samples and negative controls for the CD63/CD81 staining (panel B) or the TF staining (panel C).

Quantification of exofacial thiols was also performed as previously described [18] except that a different protocol was used for the detachment of adherent cells before staining. EDTA was added directly to the exposure medium to a final concentration of 10 mM. After incubating for 10 min at 37°C, cells were gently detached using the pipette tip and transferred to 1.5 ml centrifuge tubes.

### 2.5. Electron Spin Resonance Spectroscopy

Electron spin resonance (ESR) spectroscopy was used to determine the generation of reactive oxygen species (ROS) as described previously [31]. In brief, PM_2.5_ or PM_10_ filter pieces of 0.5 *cm* × 2 *cm* were placed in a tissue cell (ER 162 TC-Q, Bruker BioSpin GmbH, Rheinstetten, Germany) and saturated with a solution of 70 *μ*l of 40 mM Tris-HCl buffer supplemented with 100 mM of the spin trap 5,5-dimethyl-1-pyrroline N-oxide (DMPO) and 13 *μ*M ascorbate. After sealing, the tissue cell with filter material was immediately placed in the resonator of the ESR spectrometer. ESR spectra were recorded at room temperature on a Bruker EMX 1273 spectrometer equipped with an ER 4119HS high-sensitivity resonator and 12 kW power supply operating at X band frequencies. The modulation frequency of the spectrometer was 100 kHz. Instrumental conditions were as follows: center of magnetic field, 3490 G; scan range, 60 G; modulation amplitude, 1 G; receiver gain, 1 × 10^4^; microwave frequency, 9.85 GHz; power, 50 mW; time constant, 40.96 ms; scan time, 20.97 s; and number of scans, 100. Spectra were quantified (in arbitrary units) by peak surface measurements using the WIN-EPR spectrum manipulation program.

### 2.6. Data Analysis

All values are expressed as percentages of the untreated control condition. Data were analysed using GraphPad Prism V5.03. All datasets were tested for normal distribution using the Kolmogorov-Smirnov test. For those datasets that passed the Kolmogorov-Smirnov test, a one sample *t*-test was used to test whether the mean of each measurement significantly differed from the control (100%). For those datasets that were not normally distributed, the Wilcoxon signed rank test was used. To test whether NAC significantly modified the effect of each exposure, a *t*-test was applied to compare the effect of treatment only to the treatment plus NAC. *p* values < 0.05 were considered statistically significant. All EV measurements are displayed on a Log10 scale, whereas cell metabolic activity, cell surface thiols, and ESR measurements are displayed on a linear scale.

## 3. Results

### 3.1. Effect of CSE on the EV Release by Different Cell Types

**Figure 1:**
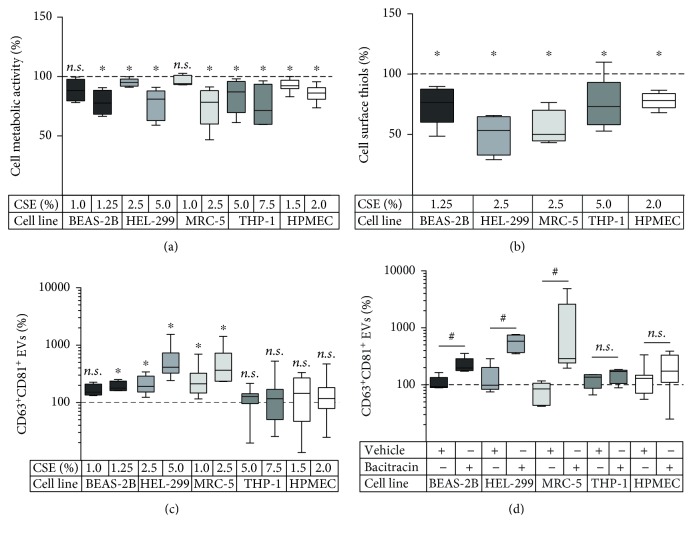
Effect of CSE and the thiol scavenger bacitracin on different pulmonary cell types. Effect of CSE exposure on (a) cell metabolic activity as determined by the MTT assay, *n* = 5‐11; (b) cell surface thiols, *n* = 4‐6; (c) release of CD63^+^CD81^+^ EVs determined by bead-based flow cytometry, *n* = 6‐13; (d) effect of 2.5 mM bacitracin on the release of CD63^+^CD81^+^ EVs, *n* = 5‐9. The box and whisker plots show the median (line in the box), 25th and 75th percentiles (outer lines of the box), and minimal and maximal values (whiskers). ^∗^ *p* < 0.05 compared to the untreated control (100%), ^#^ *p* < 0.05 for bacitracin compared to the vehicle control. Cell lines: BEAS-2B: bronchial epithelial cells; HEL-299 and MRC-5: pulmonary fibroblast cell lines; THP-1: monocyte-derived macrophages; HPMEC: human pulmonary microvascular endothelial cell line.

**Figure 2:**
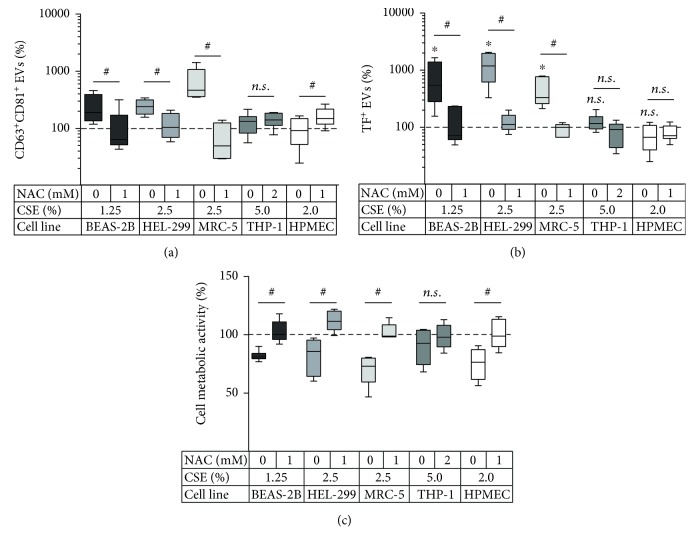
Effect of NAC on the EV release and cell metabolic activity during CSE exposure. Cells were exposed to CSE in the presence or absence of the thiol antioxidant NAC, and different readouts were performed. Concentration of (a) CD63^+^CD81^+^ EVs (*n* = 5‐9) and (b) CD63/CD81/CD9^+^TF^+^ EVs (*n* = 5‐6) as determined by bead-based flow cytometry. (c) Cell metabolic activity as determined by the MTT assay (*n* = 5‐11). The box and whisker plots show the median (line in the box), 25th and 75th percentiles (outer lines of the box), and minimal and maximal values (whiskers). ^∗^ *p* < 0.05 compared to the unexposed control (100%); ^#^ *p* < 0.05 for CSE alone compared to CSE with NAC. Cell lines: BEAS-2B: bronchial epithelial cells; HEL-299 and MRC-5: pulmonary fibroblast cell lines; THP-1: monocyte-derived macrophages; HPMEC: human pulmonary microvascular endothelial cell line.

The first aim of this study was to investigate the effect of CSE on the EV release by different pulmonary cell types. Bronchial epithelial cells (BEAS-2B), pulmonary fibroblasts (HEL-299, MRC-5), monocyte-derived macrophages (THP-1), and pulmonary microvascular endothelial cells (HPMEC-ST1.6R, short HPMEC) were exposed to CSE for 24 h. According to the MTT assay, the used CSE concentrations decreased cell metabolic activity by no more than 25% (Figure 1(a)). We then assessed the effect of these CSE concentrations on cell surface thiols and on the release of CD63^+^CD81^+^ EVs. Despite a significant CSE-induced decrease in cell surface thiols for all cell types (Figure 1(b)), a concentration-dependent increase in EV release was only observed in BEAS-2B, HEL-299, and MRC-5 cells, but not in THP-1 cells or HPMEC (Figure 1(c)). Likewise, the membrane impermeable thiol-blocking agent bacitracin was able to induce EV release by BEAS-2B, HEL-299, and MRC-5 cells, whereas no significant increase in EV release was observed for bacitracin-stimulated THP-1 and HPMEC cells (Figure 1(d)). Moreover, the thiol-antioxidant NAC inhibited the CSE-dependent EV induction in BEAS-2B, HEL-299, and MRC-5 cells, while it did not influence EV release by THP-1 cells and even slightly increased EV release in HPMEC cells (Figure 2(a)). Finally, we measured the quantity of the procoagulant protein TF on EVs released by the different cell types in response to CSE exposure in the presence or absence of NAC. EVs from BEAS-2B, HEL-299, and MRC-5 cells, but not THP-1 cells and HPMEC, showed an increased expression of TF when exposed to CSE, and this was preventable by NAC (Figure 2(b)). NAC was able to restore the cell metabolic activity during CSE exposure to approximately 100% for all cell types (Figure 2(c)).

Taken together, CSE enhanced the release of CD63^+^CD81^+^ EVs and TF^+^ EVs in bronchial epithelial cells and pulmonary fibroblasts, which was associated with a decrease in cell surface-exposed thiols. In these cells, the membrane impermeable thiol-blocking agent bacitracin also increased the EV release. Moreover, the CSE-induced release of CD63^+^CD81^+^ and TF^+^ EVs was preventable by the thiol-antioxidant NAC. In contrast, despite a significant depletion of cell surface thiols upon treatment with CSE, HPMEC and THP-1 cells did not show an enhanced EV release. In these cells, EV release could neither be triggered by bacitracin, nor was it decreased by NAC.

### 3.2. Effect of Different Respiratory Exposures on the EV Release by Bronchial Epithelial Cells

**Figure 3:**
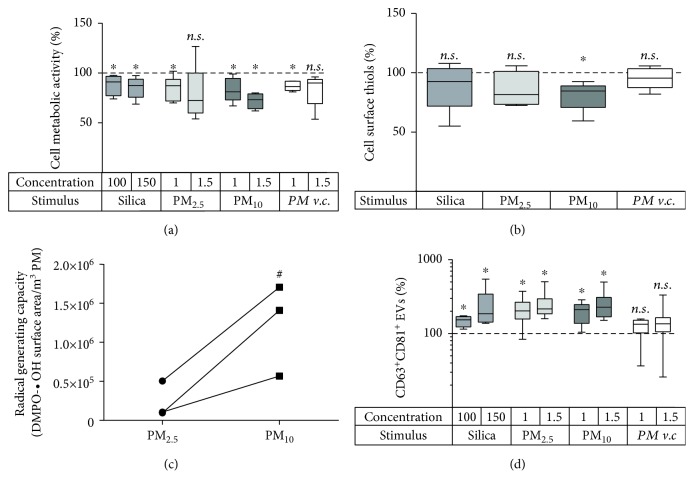
Effect of silica particles, PM_2.5_, PM_10_, and the PM vehicle control (v.c.) on bronchial epithelial cells. BEAS-2B cells were exposed for 24 h to different concentrations of the 4 stimuli. Silica concentrations are given in ×10^6^ *μ*m^2^/cm^2^, and PM concentrations in m^3^/ml. (a) Cell metabolic activity determined by the MTT assay, *n* = 5‐8; (b) cell surface thiols after 24 h exposure to 100 × 10^6^ *μ*m^2^/cm^2^ silica or 1.5 m^3^/ml PM, *n* = 5. (c) ROS-generating capacity of PM_2.5_ and PM_10_ as determined by ESR. Paired PM_2.5_ and PM_10_ samples from the same sampling location are connected by a line. (d) Release of CD63^+^CD81^+^ EVs determined by bead-based flow cytometry, *n* = 6‐9. ^∗^ *p* < 0.05 compared to the unexposed control (100%); ^#^ *p* < 0.05 for PM_10_ compared to PM_2.5_.

**Figure 4:**
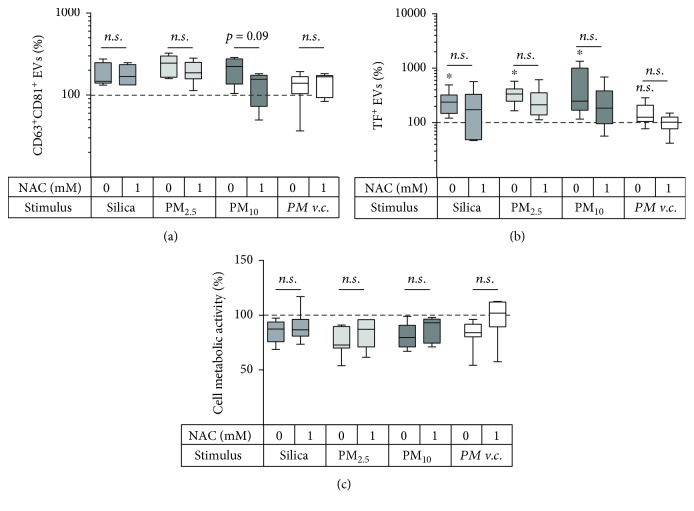
Effect of 1 mM NAC on EV release and cell metabolic activity during the exposure of BEAS-2B cells to silica (100 × 10^6^ *μ*m^2^/cm^2^) or PM (1.5 m^3^/ml). Release of (a) CD63^+^CD81^+^ EVs, *n* = 5‐9 and (b) CD63/CD81/CD9^+^TF^+^ EVs, *n* = 6‐7. (c) Cell metabolic activity as determined by the MTT assay, *n* = 5‐8. The box and whisker plots show the median (line in the box), 25th and 75th percentiles (outer lines of the box), and minimal and maximal values (whiskers). ^∗^ *p* < 0.05 compared to the unexposed control (100%).

The second aim of this study was to investigate whether respiratory exposures such as crystalline silica particles or PM from air pollution affect the EV release by BEAS-2B bronchial epithelial cells similarly to CSE. Firstly, two concentrations of silica as well as PM_2.5_ and PM_10_ were determined that decreased cell metabolic activity by at most 25% (Figure 3(a)). The concentration of cell surface thiols was not affected by silica or PM_2.5_ but was significantly decreased upon exposure to PM_10_ (Figure 3(b)). To test whether the difference between PM_2.5_ and PM_10_ could be attributed to differences in their ROS-forming capacity, ROS generation for both PM types was assessed using ESR. PM samples were derived from three different sampling locations, and for each location, PM_10_ had a higher ROS-generating capacity than PM_2.5_ (Figure 3(c)). All three exposure types (silica, PM_2.5_, and PM_10_), but not the vehicle control for PM, triggered an increase in CD63^+^CD81^+^ EVs (Figure 3(d)). NAC did not decrease the quantity of CD63^+^CD81^+^ EVs induced by silica or PM_2.5_, but a trend for a decrease was observed for PM_10_ (*p* = 0.09; Figure 4(a)). Importantly, TF^+^ EVs were also induced by all three stimuli (Figure 4(b)). Yet, NAC did not significantly decrease TF^+^ EVs for any of the exposures (Figure 4(b)). NAC treatment neither led to a significant recovery of the cell metabolic activity (Figure 4(c)).

To summarise, all tested respiratory stressors, namely, CSE (Figures 1 and 2), silica particles, PM_2.5_, and PM_10_, triggered total and TF^+^ EV release by bronchial epithelial cells. The EV induction could be entirely attributed to thiol-reactive species for CSE exposure and partly for the PM_10_ exposure, whereas another mechanism appears to regulate the EV induction by PM_2.5_ and silica particles.

## 4. Discussion

TF^+^ procoagulant EVs have been implicated as active contributors to thrombosis [32, 33] as well as pulmonary inflammation [34–37]. We have previously shown that exposure of bronchial epithelial cells to CSE causes an increase in total EV release and in TF-dependent procoagulant activity of these EVs [23]. Here, we extend these findings by showing that pulmonary fibroblasts also respond to CSE by releasing increased concentrations of EVs that express the exosome markers CD63 and CD81 as well as TF. Yet, no increased release of these EV populations was observed for monocyte-derived macrophages or pulmonary microvascular endothelial cells when stimulated with CSE. Moreover, the quantity of TF^+^ EVs released by unexposed cells was lower for endothelial cells and macrophages than for epithelial cells and fibroblasts (data not shown). This suggests that structural tissue cells may be the major source of TF^+^ EVs *in vivo*. Yet, others have reported that monocytes, macrophages, and endothelial cells respond to either CSE or PM exposure by releasing an increased quantity of TF^+^ EVs [19, 38, 39]. These divergent results may arise from the use of different cells (e.g., human umbilical vein endothelial cells [19] instead of pulmonary microvascular endothelial cells) and/or cell culture conditions. Another likely explanation is the use of different methods for EV detection. While we detected TF^+^ EVs that were enriched in exosome markers (CD63/CD81/CD9), the other studies based their findings on techniques that favour the detection of microvesicles. Thus, epithelial cells and fibroblasts may secrete TF in exosomes in response to CSE whereas macrophages and endothelial cells may secrete it in microvesicles. Side-by-side comparisons of exosome and microvesicle release by each cell type are required to investigate whether this is the case.

In the second part of this study, we investigated the effect of silica nanoparticles as well as small (PM_2.5_) and larger (PM_10_) particulates from outdoor air pollution on the release of EVs by bronchial epithelial cells. All three respiratory stressors triggered the release of CD63^+^CD81^+^ and TF^+^ EVs. Yet, they did not (silica, PM_2.5_) or only mildly (PM_10_) deplete cell surface thiols. Moreover, their effect on EV induction was not preventable by the antioxidant NAC (silica, PM_2.5_) or the prevention did not reach statistical significance (PM_10_, *p* = 0.09). This suggests that the EV induction by silica nanoparticles and PM_2.5_ is not mediated by oxidative thiol depletion and the EV induction by PM_10_ at most partly. A stronger contribution of oxidative mechanisms for PM_10_ is supported by the observation that our PM_10_ samples all had more radical-generating capacity than the paired PM_2.5_ samples.

Although PM has previously been shown to induce the release of TF^+^ microvesicles by endothelial cells *in vitro* [19], this is to our knowledge the first study reporting PM-induced EV release by epithelial cells. It is also the first study to report PM-induced release of TF on EVs that express exosome marker proteins and the first study to report that silica nanoparticles stimulate EV release. The finding that environmental as well as occupational air pollutants trigger the release of TF^+^ EVs is important as both types of exposures are associated with an increased risk for pulmonary [4–7] and cardiovascular disease [11–13] and exposure often cannot be avoided by at-risk individuals. Additional research should be performed to elucidate the cellular mechanisms that mediate the increased EV release in response to particulates, as this could allow identifying strategies for modulating particulate-induced changes in EV signalling.

This study was performed *in vitro* with immortalized cell lines and therefore has several inherent limitations. First, it is nearly impossible to accurately estimate which components of complex inhaled toxicants reach the respective target cells during real-life exposure and at what concentrations. Second, immortalized cell lines often exhibit a shift in dose response compared to primary cells. Third, cells underwent a single 24 h exposure, whereas real-life exposure to respiratory toxicants is usually chronic and/or repetitive over several years or even a lifetime. Therefore, the exposure conditions in this study cannot be directly compared to real-life exposures. Epidemiological and controlled animal or human exposure studies would be of great value to determine whether respiratory toxicants induce CD63^+^CD81^+^ and TF^+^ EVs *in vivo*. The assessment of EV concentrations in the lung (e.g., bronchoalveolar lavage fluid) and blood specimen could be complemented by the detection of cell type-specific EV surface markers to estimate the relative contribution of different cell types to the *in vivo* EV pool.

## 5. Conclusions

In this study, we demonstrated that some pulmonary cell types (i.e., epithelial cells and fibroblasts), but not all, respond to CSE by releasing CD63^+^CD81^+^ and TF^+^ EVs. Moreover, CD63^+^CD81^+^ and TF^+^ EV induction in bronchial epithelial cells appears to be a universal response to various respiratory toxicants. Clinical studies are required to determine if these EV populations are associated with (1) the exposure to respiratory toxicants *in vivo* and (2) an elevated risk for cardiovascular or pulmonary disease development. If so, TF^+^ EVs might become useful biomarkers of exposure and/or risk and may help to guide preventive treatment decisions.

## Data Availability

All data used to support the findings of this study are included within the article. Raw data used to generate the figures are available from the corresponding author upon request.

## Supplementary Materials

Supplementary Materials Supplementary Figure 1: raw data of EV detection by bead-based flow cytometry. Representative flow cytometry plots are shown for an unconditioned medium control and conditioned medium samples of unexposed and CSE-exposed BEAS-2B cells. (A) Shows the gating strategy for the bead population. (B) Shows the specificity of fluorescence signal for the detection of the total EV population stained by antiCD63-coated beads and a PE-labelled anti-CD81 detection antibody, compared to a PE-labelled isotype control. (C) Shows the specificity of the fluorescence signal for the detection of the TF-positive EV population stained by beads coated with a mixture of anti-CD63, anti-CD81, and anti-CD9 antibodies and a PE-labelled anti-TF detection antibody, compared to a PE-labelled isotype control. Supplementary Table 1: overview of materials with the respective providers and catalogue numbers. Supplementary Table 2: table for unit conversion for the PM exposure. PM samples were collected at 3 different locations with different levels of air pollution. To reflect the real-life exposure at these locations, PM exposure was standardized per volume of filtered air rather than per weight of PM.
