# Supplementary material for: Cell Type- and Exposure-Specific Modulation of CD63/CD81-Positive and Tissue Factor-Positive Extracellular Vesicle Release in response to Respiratory Toxicants
Source: Oxid Med Cell Longev. 2019 Aug 14;2019:5204218. doi: 10.1155/2019/5204218 (PMC6710792; doi:10.1155/2019/5204218)
Supplement: Supplementary Materials — Supplementary Figure 1: raw data of EV detection by bead-based flow cytometry. Representative flow cytometry plots are shown for an unconditioned medium control and conditioned medium samples of unexposed and CSE-exposed BEAS-2B cells. (A) Shows the gating strategy for the bead population. (B) Shows the specificity of fluorescence signal for the detection of the total EV population stained by antiCD63-coated beads and a PE-labelled anti-CD81 detection antibody, compared to a PE-labelled isotype control. (C) Shows the specificity of the fluorescence signal for the detection of the TF-positive EV population stained by beads coated with a mixture of anti-CD63, anti-CD81, and anti-CD9 antibodies and a PE-labelled anti-TF detection antibody, compared to a PE-labelled isotype control. Supplementary Table 1: overview of materials with the respective providers and catalogue numbers. Supplementary Table 2: table for unit conversion for the PM exposure. PM samples were collected at 3 different locations with different levels of air pollution. To reflect the real-life exposure at these locations, PM exposure was standardized per volume of filtered air rather than per weight of PM. [file 5204218.f1.pdf]

Supplementary table 1 Overview of materials with the respective providers and catalogue numbers

Product	Provider	Catalogue number
BEAS-2B cells	ATCC	CRL-909
MRC-5 cells	ATCC	CCL-171
HEL-299 cells	ATCC	CCL-137
THP-1 cells	ATCC	TIB-202
HPMEC-ST1.6R cells	Dr. C.J. Kirkpatrick lab, Institute for Pathology, University of Mainz, Germany	-
MycoAlert™ Mycoplasma Detection Kit	Lonza	LT07-118
Minimal Essential Medium (MEM), no glutamine	Gibco	21090
Minimal Essential Medium Non-Essential Amino Acids (100X Solution)	MP Biomedicals LLC	1681049
L-Glutamine (200mM)	Thermo Fisher Scientific	25030081
Fetal calf serum (FCS)	Biowest	S181G
RPMI 1640	Gibco	52400
β-mercaptoethanol	Sigma-Aldrich	M3148
Sodium pyruvate (100 mM)	Thermo Fisher Scientific	11360070
Glucose	Sigma-Aldrich	G7021
MCDB 131 medium, no glutamine	Thermo Fisher Scientific	10372019
Hydrocortisone	Sigma-Aldrich	H0396
human recombinant epidermal growth factor	Sigma-Aldrich	11376454001
Penicillin-Streptomycin (10,000 U/mL)	Gibco	15140122
Gelatin from bovine skin Type B	Sigma-Aldrich	G9391
24-well plates	Corning	3524
12-well plates	Corning	3513
Phorbol 12-myristate 13-acetate (PMA)	Sigma-Aldrich	P8139
DMEM/F-12, HEPES, no phenol red	Gibco	11039
Bacitracin	Sigma-Aldrich	11702
N-acetyl-L-cysteine	Sigma-Aldrich	A9165
research reference cigarette	University of Kentucky	3R4F
Dichloromethane	Sigma-Aldrich	270997
PBS	Gibco	10010023
Silica nanoparticles	C&E Mineral Corp, King of Prussia, PA	-
Thiazolyl Blue Tetrazolium Bromide 98% (MTT)	Sigma-Aldrich	M2128
4 µm aldehyde/sulphate latex beads 4% (w/v)	Thermo Fisher Scientific	A37304
mouse anti-human CD81 Clone JS-81	BD Biosciences	555675
mouse anti-human CD63 clone H5C6	BD Biosciences	556019
mouse anti-human CD9 clone M-L13	BD Biosciences	555370
MES buffer	Sigma-Aldrich	M1317
phycoerythrin (PE)-labelled mouse anti-human TF/CD142 antibody	BD Biosciences	550312
phycoerythrin (PE)-labelled mouse anti-human CD81 antibody	BD Biosciences	555676
phycoerythrin (PE)-labelled mouse IgG1-k isotype control	BD Biosciences	555749
Ethylenediaminetetraacetic acid disodium salt (EDTA) dihydrate	Sigma-Aldrich	E5134
Alexa-Fluopr 488 maleimide	Thermo Fisher Scientific	A10254
5,5-Dimethyl-1-pyrroline N-oxide (DMPO)	Sigma-Aldrich	D5766
Ascorbate	Sigma-Aldrich	11140

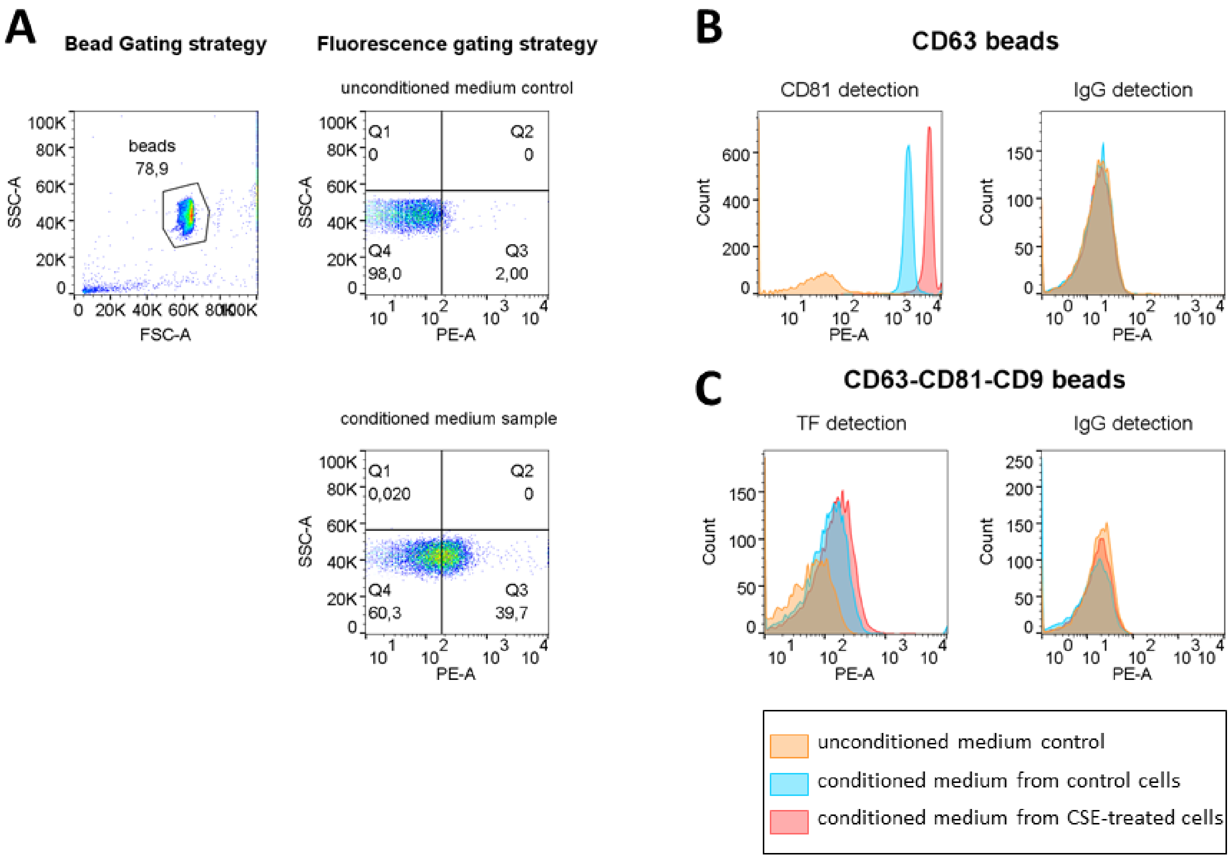

Supplementary Figure 1 Raw data of EV detection by bead-based flow cytometry. Representative flow cytometry plots are shown for an unconditioned medium control and conditioned media samples of unexposed and CSE-exposed BEAS-2B cells. (A) Shows the gating strategy for the bead population. (B) Shows the specificity of fluorescence signal for the detection of the total EV population stained by anti-CD63 coated beads and a PE-labelled anti-CD81 detection antibody, compared to a PE-labelled isotype control. (C) Shows the specificity of the fluorescence signal for the detection of the TF-positive EV population stained by beads coated with a mixture of anti-CD63, anti-CD81 and anti-CD9 antibodies and a PE-labelled anti-TF detection antibody, compared to a PE-labelled isotype control.

Supplementary table 2 Table for unit conversion for the PM exposure. PM samples were collected at 3 different locations with different levels of air pollution. To reflect the real life exposure at these locations, PM exposure was standardized per volume of filtered air rather than per weight of PM.

PM sample	µg PM/m^3	cm^2 cell surface area / ml of culture medium	1 m^3/ml in µg/cm^2	1.5 m^3/ml in µg/cm^2
PM2.5 n1	50.3	3.4	13.2	19.8
PM2.5 n2	30.4		8.0	12.0
PM2.5 n3	30.8		8.1	12.2
PM10 n1	118.1		31.1	46.6
PM10 n2	71.25		18.8	28.1
PM10 n3	77.1		20.3	30.4
